# Approach to the patient with eosinophilia in the era of tyrosine kinase inhibitors and biologicals

**DOI:** 10.1007/s11899-024-00738-7

**Published:** 2024-07-22

**Authors:** Johannes Lübke, Georgia Metzgeroth, Andreas Reiter, Juliana Schwaab

**Affiliations:** Department of Hematology and Oncology, University Hospital Mannheim, Heidelberg University, Theodor-Kutzer-Ufer 1-3, 68167 Mannheim, Germany

**Keywords:** Eosinophilia, Hypereosinophilia, MLN-TK, CEL, HES

## Abstract

**Purpose of Review:**

In this review, we aim to explore the optimal approach to patients presenting with eosinophilia, considering recent advances in diagnostic and therapeutic strategies. Specifically, we focus on the integration of novel therapies into clinical practice to improve patient outcomes.

**Recent Findings:**

Advanced insights into the clinical and genetic features of eosinophilic disorders have prompted revisions in diagnostic criteria by the World Health Organization classification (WHO-HAEM5) and the International Consensus Classification (ICC). These changes reflect a growing understanding of disease pathogenesis and the development of targeted treatment options. The therapeutic landscape now encompasses a range of established and novel therapies. For reactive conditions, drugs targeting the eosinophilopoiesis, such as those aimed at interleukin-5 or its receptor, have demonstrated significant potential in decreasing blood eosinophil levels and minimizing disease flare-ups and relapse. These therapies have the potential to mitigate the side effects commonly associated with prolonged use of oral corticosteroids or immunosuppressants. Myeloid and lymphoid neoplasms with eosinophilia and tyrosine kinase (TK) gene fusions are managed by various TK inhibitors with variable efficacy.

**Summary:**

Diagnosis and treatment rely on a multidisciplinary approach. By incorporating novel treatment options into clinical practice, physicians across different disciplines involved in the management of eosinophilic disorders can offer more personalized and effective care to patients. However, challenges remain in accurately diagnosing and risk-stratifying patients, as well as in navigating the complexities of treatment selection.

## Introduction

**Fig. 1:**
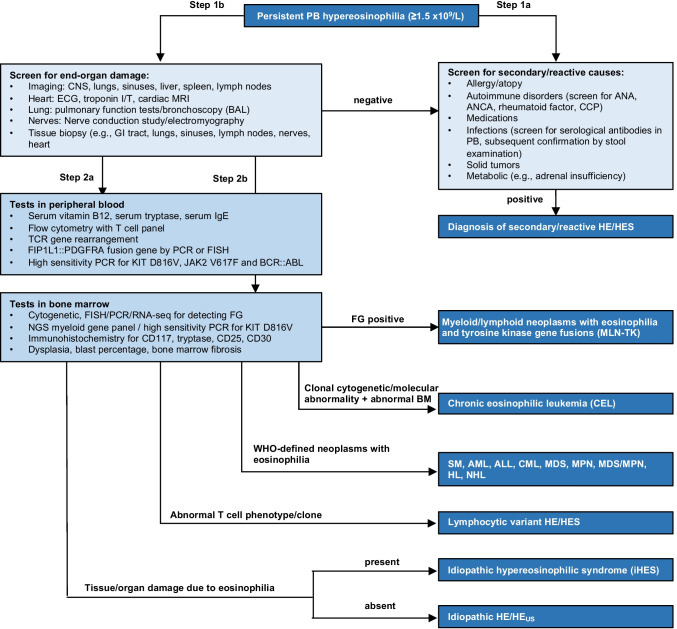
Diagnostic approach to the patient with eosinophilia. Abbreviations: ALL, acute lymphoid leukemia; AML, acute myeloid leukemia; ANA, antinuclear antibodies; ANCA, antineutrophil cytoplasmic antibodies; BAL, bronchoalveolar lavage; BM, bone marrow; CCP, cyclic citrullinated peptide; CEL, NOS; chronic eosinophilic leukemia, not otherwise specified; CML, chronic myeloid leukemia; CNS, central nervous system; ECG, electrocardiogram; FG, fusion gene(s); FISH, fluorescence in situ hybridization; GI, gastrointestinal; HE, hypereosinophilia; HES, hypereosinophilic syndrome; HE_us_, hypereosinophilia with undetermined significance; HL, Hodgkin’s lymphoma; IgE immunoglobulin E; iHES, idiopathic hypereosinophilic syndrome; MDS, myelodysplastic neoplasm; MDS/MPN, myelodysplastic/ myeloproliferative neoplasm; MLN-TK; myeloid/lymphoid neoplasms with eosinophilia and tyrosine kinase gene fusions; MPN, myeloproliferative neoplasm; MRI, magnetic resonance imaging; NGS, next-generation-sequencing; NHL, non-Hodgkin’s lymphoma; PB, peripheral blood; PCR, polymerase chain reaction; RNA-seq, RNA sequencing; TCR, T cell receptor; WHO, World Health Organization Modified from Gotlib et al. [1] and Wang et al. [2]

Eosinophilia and hypereosinophilia (HE) are defined as an absolute eosinophil count of ≥ 0.5 × 10^9^/L and ≥ 1.5 × 10^9^/L in peripheral blood (PB), with an arbitrarily distinction into mild (< 1.5 × 10^9^/L), moderate (1.5–5 × 10^9^/L), and severe (≥ 5 × 10^9^/L) eosinophilia. The underlying etiology of eosinophilia ranges from benign reactive conditions to life-threatening neoplasms and its correct attribution is often challenging. After confirmation of HE, the diagnostic workup should progress in a stepwise manner, first by excluding the most frequent secondary/reactive causes (e.g., allergies, infections but also autoimmunopathies such as eosinophilic granulomatosis with polyangiitis [EGPA]), followed by evaluating potential primary/neoplastic causes (e.g., myeloid/lymphoid neoplasms with eosinophilia and tyrosine kinase gene fusions [MLN-TK], chronic eosinophilic leukemia [CEL], eosinophilia associated with other myeloid neoplasms), and ultimately by confirming or excluding the diagnosis of lymphocytic (L-HES) or idiopathic hypereosinophilic syndrome (iHES, Fig. 1).

Recent advances in our understanding of HE related clinical and genetic features have led to significant revisions in diagnostic criteria by the World Health Organization classification (WHO-HAEM5) and the International Consensus Classification (ICC), reflecting a growing appreciation of the complex (genetic) pathogenesis underlying different eosinophilic disorders [3, 4].

In this manuscript, we aim to provide a comprehensive overview of the expanding therapeutic landscape, encompassing both established treatment modalities and innovative therapeutic agents of which some are currently undergoing evaluation in clinical trials. Through a comprehensive examination of recent developments and emerging trends in the field, we try to offer insights into the management of eosinophilic disorders that will inform clinical decision-making and improve patient outcomes.

### Secondary/reactive eosinophilia

Eosinophilia is predominantly of secondary/reactive origin with a multitude of different causes (Table 1). Globally, parasitic infections and drug reactions to medications are among the most common causes for eosinophilia. Furthermore, mild to moderate PB eosinophilia is associated with several autoimmune conditions. EGPA is classified as a small-vessel, anti-neutrophil cytoplasmic antibody (ANCA)-associated vasculitis (AAV) [5, 6]. However, only approximately 30–47% of EGPA patients test positive for ANCA, predominantly showing anti-myeloperoxidase (MPO) ANCA positivity [7]. Recent studies have revealed that ANCA-positive and -negative EGPA are linked to distinct genetic polymorphisms, suggesting different underlying pathogenic mechanisms [8]. [9–13]. Patients with ANCA-positive EGPA are more likely to develop a vasculitic phenotype, whereas patients without ANCA are more predisposed to eosinophilic end organ damage. However, ANCA status alone is insufficient to predict the individual clinical presentation or guide treatment decisions. Oral corticosteroids are the standard of care [6]. In cases with end-organ involvement or life-threatening symptoms, corticosteroids are administered along with immunosuppressive drugs (e.g., cyclophosphamide, azathioprine or rituximab) [6]. Most recently, the MIRRA trial (NCT02020889) demonstrated that approximately half of the participants with relapsing or refractory EGPA who were treated with the anti-interleukin (IL)-5 monoclonal antibody mepolizumab had clinically relevant improvements in the rates of protocol-defined remission (28% vs. 3% for placebo) and relapse (annualized relapse rate 1.14 vs. 2.27 for placebo), and were able to reduce the dose of steroids (< 4.0 mg prednisolone per day, 44% vs. 7% for placebo) compared to placebo [14].

**Table 1:** Non-exhaustive list of possible secondary/reactive causes for eosinophilia

• Drugs (e.g., antiepileptics, antibiotics, Drug reaction with eosinophilia and systemic symptoms, *most common cause in industrialized countries*)
• Allergic disorders (e.g., asthma, atopic dermatitis/eczema, seasonal allergic disorders)
• Rheumatological disease (e.g., eosinophilic granulomatosis with polyangiitis, systemic lupus erythematosus, rheumatoid arthritis, and eosinophilic fasciitis)
• Pulmonary disease (e.g., Loffler syndrome, sarcoidosis)
• Gastrointestinal disorders (e.g., primary gastrointestinal eosinophilic disorders including eosinophilic esophagitis and chronic pancreatitis)
• Dermatological disorders (e.g., Wells syndrome, angiolymphoid hyperplasia)
• Neoplasms (non‐hematologic and hematologic: e.g., T‐cell lymphomas, Hodgkin lymphoma, systemic mastocytosis, solid tumors)
• Parasitic infections (e.g., helminth infections, *most common cause worldwide*)

Modified from Larsen et al. [15]

Neoplastic conditions, both hematologic (e.g., systemic mastocytosis, acute leukemias, myeloproliferative neoplasms, lymphoma) and non-hematologic (e.g., solid tumors), can also trigger eosinophilia.

Administered medications (e.g., corticosteroids) and transient medical conditions (e.g., bacterial infections) may temporarily lower the absolute eosinophil count, masking the real degree of eosinophilia. If secondary causes of eosinophilia have been excluded or are unlikely, and the cause of eosinophlia remains unclear, screening for a primary eosinophilic disorder is necessary, ideally before a potential end-organ damage occurs.

### Myeloid/lymphoid neoplasms with eosinophilia and tyrosine kinase gene fusions (MLN-TK)

According to the 2022 WHO/ICC classification, the term “myeloid/lymphoid neoplasms with eosinophilia and rearrangement of *PDGFRA*, P*DGFRB*, or *FGFR1*, or with *PCM1::JAK2*” has been replaced with MLN-TK to specify the underlying molecular genetic changes and to include cases with *ETV6::ABL1*, *FLT3* fusions, or other tyrosine kinase (TK) gene fusions [2, 4, 16]. Nearly 100 different TK fusion genes, involving at least six TK (*PDGFRA*, *PDGFRB*, *FGFR1*, *JAK2*, *ABL1*, *FLT3*), have been identified in distinct MLN with or without eosinophilia [17, 18]. A comprehensive overview of the most common fusion partners is provided in Table 2. Besides eosinophilia, patients often present with monocytosis and an elevated serum tryptase, particularly in cases with *PDGFRA* or *PDGFRB* fusion genes [18, 19]. Elevated vitamin B12 levels are a common, yet non-specific marker for myeloproliferative neoplasms in general.

**Table 2:** Genetic abnormalities, clinical manifestation and specific treatment approaches for myeloid/lymphoid neoplasms with eosinophilia and tyrosine kinase gene fusions (MLN-TK) as per 2022 World Health Organization classification and International Consensus Classification [3, 4]

TK gene	Most common fusion	Analyses	Other partner genes/ variants	Typical clinical and BM manifestation	Accompanying mutations	Targeted therapy	Prognosis
*PDGFRA*	Cryptic deletion 4q12/ *FIP1L1:: PDGFRA*	FISH, RT-PCR	*CDK5RAP2; STRN; KIF5B; TNKS2; ETV6, BCR*	Common: CEL-like BM, extramedullary involvement Others: B-ALL (in children), AML or mast cell proliferations	20–50%: *ASXL1, BCOR, DNMT3A, RUNX1, SRSF2, TET2*	Imatinib, may be terminated	Durable and complete hematologic, cytogenetic and molecular remission
*PDGFRB*	t(5;12)(q32;p13.2)/ *ETV6::PDGFRB*	Karyotype, FISH, RT-PCR	> 30 partners, some cryptic	Common: CEL-like or monocytosis with eosinophilia Others: ALL, AML or mast cell proliferations	30–50%: *ASXL1, BCOR, DNMT3A, NRAS, STAG2, STAT5B, TET2, ZSRS2*	Imatinib, may be terminated	Durable and complete hematologic, cytogenetic and molecular remission
*FGFR1*	t(8;13)(p11.2;q12.1)/ *ZMYM2::FGFR1*	Karyotype, FISH, RT-PCR	15 other partners including *BCR*	Common: Extramedullary sites, T-ALL with BM MPN-like or blast phase of MPN; Others: B-ALL, myeloid sarcoma, AML or MPAL	70–80%: *RUNX1, ASXL1, CSFR3, STAG2*	Pemigatinib (anti-FGFR1-3), Futibatinib (anti-FGFR1-4)	Pemigatinib: Complete clinical and cytogenetic response, more durable in chronic vs. blast phase, consider alloHCT
*JAK2*	t(8;9)(p22;p24.1)/ *PCM1::JAK2*	Karyotype, FISH	*ETV6* and *BCR*	Common: MPN or MDS/MPN like BM with eosinophilia Others: B- and T-ALL with BM MPN	14–50%: *ASXL1, BCOR, BCORL1, CD36, EP300, ETV6, RUNX1, SRSF2, TET2, TP53*	Ruxolitinib	Hematologic and cytogenetic response, not durable, consider alloHCT
*FLT3*	t(12;13)(p13.2;q12.2)/ *ETV6::FLT3*	Karyotype, FISH, RT-PCR	*ZMYM2, TRIP11, SPTBN1, GOLGB1, CCDC88C, MYO18A, BCR*	T-ALL or myeloid sarcoma with CEL-like or MDS/MPN BM features	~ 50%: *ASXL1, RUNX1, STAT5B, SRSF2, TET2, TP53, U2AF1*	Sorafenib, Sunitinib, Midostaurin, Gilteritinib	Various hematologic and cytogenetic responses
*ETV6::ABL1*	t(9;12)(q34.1;p13.2)/ *ETV6::ABL1*	FISH, RT-PCR	Unknown	CML-like with frequent eosinophilia in chronic or blast phase	40–50%: *ARID2, CDKN1B, TP53, SMC1A*	Dasatinib, Nilotinib (Imatinib)	Variable durable hematologic and cytogenetic response

Abbreviations: ALL, acute lymphoid leukemia; alloHCT, allogeneic stem cell transplantation; AML, acute myeloid leukemia; BM, bone marrow; CEL, chronic eosinophilic leukemia; CML, chronic myeloid leukemia; FISH, fluorescence in situ hybridization; MDS/MPN, myelodysplastic/ myeloproliferative neoplasm; MPAL, mixed-phenotype acute leukemia; MPN, myeloproliferative neoplasms; RT-PCR, reverse transcription-polymerase chain reaction; TK, tyrosine kinase

Modified from Tzankov et al. [20] and Wang et al. [2]

The blast phase in the bone marrow (BM) or at extramedullary sites (extramedullary disease, EMD), which is often initially diagnoses as myeloid sarcoma or high-grade (T-/B-cell) lymphoma without knowledge of an underlying TK fusion gene, may be present at diagnosis (primary blast phase) or develops in due course (secondary blast phase) [21]. Patients should undergo imaging (e.g., computed tomography) to check for extramedullary manifestation. In a recently published register based study on 135 MLN-TK patients, primary or secondary blast phase manifested with similar frequency either in the BM or as EMD in 28% of patients, of which 61% were of myeloid and 39% of lymphoid origin [18]. Primary or secondary blast phase in the BM was equally prevalent (each 50%), whereas primary EMD was more common (83%) than secondary EMD (17%). A discordance between myeloid and lymphoid lineage involvement in BM and at extramedullary sites was regularly seen in 50% of patients. Patients with *PDGFRA* and *PDGFRB* fusion genes (16%) were less likely to exhibit primary blast phase compared to those with *FGFR1*, *JAK2*, and *ETV6::ABL1* fusion genes (26%). Secondary blast phase was also only observed in 6% of patients with *PDGFRA* and *PDGFRB* fusion genes after a median of 87 months. In addition to eosinophilia, BM morphology in MLN-TK frequently displayed an increase of mast cells and fibrosis [22]. In cases with dense mast cell aggregates and/or an elevated serum tryptase, molecular studies with high sensitivity (e.g., digital PCR) should be conducted to rule out a *KIT* D816V mutation for systemic mastocytosis [22–27]. The detection of fusion genes may be challenging due to variable clinical presentation, cryptic fusions or unknown fusion partners. Therefore, integrating conventional molecular- and cytogenetic analysis, along with advanced sequencing technologies such as RNA sequencing or next-generation-sequencing (NGS), might be necessary.

Considering the risks of both irreversible end-organ damage associated with persistent HE and possible transformation into blast phase with poor prognosis, it is recommended to initiate treatment immediately irrespective of the clinical symptoms [28]. Potentially effective targeted treatment with tyrosine kinase inhibitors (TKI) exist for all individual TK fusion genes. Imatinib has been proven to be highly effective in MLN with *PDGFRA* and *PDGFRB* fusion genes, with achievement of durable complete hematologic, cytogenetic (e.g., *PDGFRB* fusion genes) and molecular (e.g., *FIP1L1::PDGFRA*) remissions in more than 90% of patients. Patients are initially treated with a daily dose of 100–400 mg. Maintenance treatment with 100 mg three times per week is sufficient for complete molecular remissions [29–40]. Retrospective studies have examined the possibility of discontinuing imatinib once complete molecular remission of *FIP1L1::PDGFRA* is achieved [39, 40]. In one study involving 12 patients with the *FIP1L1::PDGFRA* fusion gene, the median time to relapse after stopping imatinib was 5.6 months, with approximately 30–40% of patients experiencing a treatment-free remission lasting longer than 3 years and a rapid second hematologic and molecular response to imatinib in those with a relapse [18]. While secondary resistance to imatinib is rare, it typically involves one of two specific mutations: *PDGFRA* T674I, which has shown in vitro response to ponatinib, or *PDGFRA* D842V, for which avapritinib, approved for gastrointestinal stromal tumors (GIST), holds promise [41–43].

In contrast, TK fusion genes with involvement of *FGFR1, JAK2* or *FLT3* are associated with a more aggressive phenotype and clinical course with variable sensitivity to TKI. *RUNX1* mutations, reflecting clonal stem cell impairment, are identifiable in the majority of patients with *FGFR1* fusion genes (70–80%) and typically correlate with a poorer prognosis [44]. The 1-year survival rate in *FGFR* fusion driven patients is approximately 40% [45]. Therefore, there is a high unmet need for effective treatment options. Pemigatinib, an oral inhibitor selective for FGFR1-3 given at an oral dose of 13.5 mg once daily, has been approved for adults with MLN-TK harboring *FGFR1* fusion genes based on data from an ongoing phase 2, open-label, multicenter trial (NCT03011372) [46]. Complete remission rate was 65%; cytogenetic response rate was 77.4%. Among patients achieving complete remission, a significant reduction in *FGFR1* fusion transcripts was observed, with 81% experiencing a > 2-log reduction and 48% a > 3-log reduction [47]. Although complete clinical and cytogenetic responses were also observed in blast phase, they were less frequent and less durable compared to chronic phase disease [46, 47]. A retinal pigment epithelial detachment (RPED) occurred in 26% of patients, with no instances of severe (grade 3–4) RPED observed. Before and during treatment, ophthalmological examinations are therefore recommended. Hyperphosphatemia was reported in 74% of patients, with severe (grade 3–4) hyperphosphatemia observed in 2.9% of cases. A low phosphate-diet and phosphate-lowering therapies should be initiated based on the severity. Overall, 80% of patients starting on the recommended dosage required dose reductions of pemigatinib due to adverse events. Depending on the severity of adverse events, initial dose reduction is recommended to 9 mg once daily, followed by subsequent reductions to 4.5 mg once daily, and 4.5 mg once daily for the first 14 days of each 21-day cycle. Pemigatinib may serve as a bridge to allogeneic stem cell transplantation (alloHCT) in these patients. In a retrospective study involving 22 patients with *FGFR1* fusions who underwent alloHCT, the estimated 5-year survival rate was 74%, with progression-free survival at 63%. The rates of non-relapse mortality and relapse were 14% and 23%, respectively [48]. The potential of combining pemigatinib with chemotherapy in transplant-ineligible patients, particularly for aggressive phenotypes, warrants further investigation, as does the use of pemigatinib as maintenance therapy following alloHCT.

Futibatinib, an oral selective small molecule inhibitor of FGFR1-4, given at an oral dose of 20 mg once daily, has been assessed in a 55-year-old male, resulting in the first reported case of complete hematologic and cytogenetic remission in an FGFR1-driven myeloid neoplasm [49]. This has led to an ongoing, phase 2, open-label, multicenter trial for transplant-ineligible patients (NCT04189445) [50].

Six distinct fusion genes involving *JAK2* are yet identified, with *PCM1::JAK2* being the most common. Treatment with ruxolitinib (usually doses of 10 mg per day BID or higher) may lead to transient remission rates, but should primarily be seen as bridge to alloHCT in fit patients [51]. Although we currently lack data, fedratinib, momelotinib, or pacritinib could potentially demonstrate efficacy. *ETV6::FLT3* constitutes about half of seven distinct *FLT3* fusion genes. Partial and frequently transient effectiveness was observed with sorafenib, sunitinib, midostaurin, and gilteritinib. AlloHCT was documented in three patients, all of whom were in complete response at the time of reporting [52–57]. While *ETV6::PDGFRB* usually nicely responds to imatinib*,* fusions like *ETV6::ABL1* and others often show lack of remission to imatinib but require nilotinib or ponatinib for achievement of remission [58]. Further treatment options are presented in Tables 2, 3.

**Table 3:** Active clinical trials as per clin-trial.gov in February 2024

Drug (NCT number) ^*a*^	Adminis-tration	Trial name	Design	Start date – estimated completion date	Estimated enrollment	Primary outcome	Locations
Benralizumab, anti-IL-5r mAb (NCT04191304)	sc	A Multicentre, Randomised, Double-blind, Parallel-group, Placebo-controlled, 24 Week Phase III Study With an Open-label Extension to Evaluate the Efficacy and Safety of Benralizumab in Patients With Hypereosinophilic Syndrome (NATRON)	Phase 3 (double-blind, comparator: placebo)	July 2020 – November 2026	120 participants (≥ 12 years)	Time to first HES worsening/flares	Austria, Belgium, Denmark, France, Germany, Israel, Italy, Japan, Netherlands, Poland, Switzerland, United States
Depemokimab, anti-IL-5 mAb (NCT05334368)	sc	A Randomized, Double-blind, Placebo-controlled Study to Investigate the Efficacy and Safety of Depemokimab in Adults With Hypereosinophilic Syndrome (HES) (DESTINY)	Phase 3 (double-blind, comparator: placebo)	September 2022 – March 2026	120 participants (≥ 18 years)	Frequency of HES flares	China, Japan, Republic of Korea, Spain, United States
Futibatinib, FGFR1-4 inhibitor (NCT04189445)	oral	A Phase 2 Study of Futibatinib in Patients With Specific FGFR Aberrations (TAS-120–202)	Phase 2 (open-label)	August 2020–June 2024	115 participants (≥ 18 years) with 20 participants with MLN-TK	Complete response as per MLN criteria	Belgium, France, Germany, Hong Kong, Italy, Japan, Netherlands, Portugal, Republic of Korea, Singapore, Spain, Sweden, Turkey, United Kingdom, United States
Imatinib, TKI; Ruxolitinib, JAK-inhibitor (NCT00044304)	oral	Efficacy of Tyrosine Kinase Inhibition in Reducing Eosinophilia in Patients With Myeloid and/or Steroid-Refractory Hypereosinophilic Syndrome	Phase 2 (open-label)	September 2002 – January 2026	60 participants (≥ 2 years for imatinib and ≥ 18 years for ruxolitinib)	Peripheral blood absolute eosinophil count	United States
Mepolizumab, anti-IL-5 mAb (NCT04965636)	sc	A Phase 3, 52-week, Open-label, Single Arm Study to Investigate the Efficacy and Safety of Mepolizumab SC in Participants Aged 6 to 17 Years With Hypereosinophilic Syndrome (SPHERE)	Phase 3 (open-label)	July 2022 – September 2024	25 participants (6–17 years)	Frequency of HES flares	Argentina, Spain, United States
Pemigatinib	oral	A Phase 2, Open-Label, Monotherapy, Multicenter Study to Evaluate the Efficacy and Safety of Pemigatinib (INCB054828) in Subjects With Myeloid/Lymphoid Neoplasms With FGFR1 Rearrangement (FIGHT-203)	Phase 2 (open-label)	April 2017 – July 2024	47 participants (≥ 18 years)	Complete response as per MLN criteria	Austria, Belgium, Canada, France, Germany, Italy, Japan, Spain, Switzerland, United Kingdom, United States
Ruxolitinib, JAK inhibitor (NCT03801434)	oral	Phase 2 Study of Ruxolitinib in Idiopathic Hypereosinophilic Syndrome and Primary Eosinophilic Disorders	Phase 2 (open- label)	November 2019 – November 2025	25 participants (≥ 18 years)	Overall hematologic response rate	United States

Abbreviations: HES, hypereosinophilic syndrome; mAb, monoclonal abbreviations; M/LN-eo-TK, myeloid/lymphoid neoplasms with eosinophilia and tyrosine kinase gene fusions; r, receptor; sc, subcutaneously; TKI, tyrosine kinase inhibitor

^***a***^ Only active clinical trials as clin-trial.gov are reported

Modified from Tzankov et al. [20] and Wang et al.[2]

### Chronic eosinophilic leukemia

CEL is a heterogenous disorder that is characterized by persistent eosinophilia while not meeting criteria for other genetically defined entities (Table 4). Diagnostic criteria further mandate abnormal BM morphology (e.g., dysplastic megakaryocytes with or without dysplastic features in other lineages or increased blasts ≥ 5% in the BM and/or ≥ 2% in the PB) as well as demonstration of a clonal cytogenetic abnormality and/or somatic mutation(s) [2, 4, 59]. Additionally, the BM typically displays significant fibrosis associated with an eosinophilic infiltrate [59]. Most of the reported mutations have been identified in genes associated with DNA methylation and chromatin modification, including *ASXL1*, *TET2*, *EZH2*, and *DNMT3A* [59–61]. However, mutations have also been observed in other genes such as *SRSF2*, *TP53*, and *SETBP1* [59, 60]. One recent study detected the *STAT5B* N642H mutation as a recurrent event (1.6%) in patients referred with a diagnosis of eosinophilia, including those who would have otherwise been diagnosed with iHES [62]. In a separate investigation conducted by the French referral center for hypereosinophilic syndromes (CEREO), 64 individuals with TK fusion-negative HE underwent screening via NGS using a customized panel comprising 149 genes to detect somatic mutations. Among these, 35 patients (54%) exhibited at least one mutation within the JAK-STAT pathway, encompassing mutations in *STAT5B* (n = 18; N642H, n = 13), *JAK1* (indels in exon13, n = 5; V658F/L, n = 2), and *JAK2* (V617F, n = 6; indels in exon 13, n = 2). Additionally, previously unreported somatic mutations were identified in *JAK2*, *JAK1*, *STAT5B*, and *STAT5A*, with three patients sharing the same *STAT5A* V707fs mutation [63]. Exclusion of systemic mastocytosis by high sensitivity molecular studies (e.g., digital PCR) is recommend. Due to the lack of specific treatment options and rapid transformation into secondary blast phase, prognosis of CEL is generally poor. In a case series of 10 patients reported by Wang et al., the median overall survival was only 22 months with 5 patients developing AML after a median of 20 months from diagnosis [64]. Consensus on the optimal frontline treatment for CEL remains elusive [21]. While corticosteroids, hydroxyurea, PEG-IFN-α, and imatinib have been employed to mitigate leukocytosis and HE, their effectiveness tends to be temporary. PEG-IFN-α has shown partial success in inducing hematologic and molecular/cytogenetic responses, along with ameliorating end-organ damage, also in patients resistant or refractory to corticosteroids and hydroxyurea [65–67]. Hematologic improvements from empiric imatinib usage, in the absence of a specific tyrosine kinase target, may primarily result from nonspecific myelosuppression. Hypomethylating agents and/or alloHCT are further treatment options.

**Table 4:** Diagnostic criteria for idiopathic hypereosinophilic syndrome as per International Consensus Classification [4]

1. Persistent peripheral blood hypereosinophilia (eosinophil count ≥ 1.5 × 10^9^/L and ≥ 10% eosinophils) ^***a***^
2. Organ damage and/or dysfunction attributable to tissue eosinophilic infiltrate ^***b***^
3. No evidence of a reactive, well-defined autoimmune disease or neoplastic condition/disorder underlying the hypereosinophilia
4. Exclusion of lymphocyte variant hypereosinophilic syndrome
5. Bone marrow morphologically within normal limits except for increased eosinophils
6. No molecular genetic clonal abnormality, with the caveat of clonal hematopoiesis of indeterminate potential (CHIP)

The diagnosis of iHES requires all 6 criteria

^***a***^ Preferably a minimal duration of 6 months if documentation is available. In patients with end-organ damage requiring prompt treatment, the diagnosis can be established after 4 weeks or with a repeated complete blood count after a minimum interval of 2 weeks

^***b***^ Hypereosinophilia of uncertain significance has no tissue damage, but otherwise fulfills the same diagnostic criteria

^***c***^ An abnormal T-cell population must be detected by flow cytometry with or without T-cell receptor clonality by molecular analysis

### Idiopathic hypereosinophilic syndrome

iHES is characterized by (i) persistent HE in PB (≥ 6 months or ≥ 2 weeks if end-organ damage necessitates immediate treatment), (ii) end-organ damage caused by eosinophilic infiltration and (iii) absence of a reactive, familial or neoplastic etiology, as well as exclusion of L-HES (Table 5) [4]. L-HES is a distinct subtype, characterized by aberrant clonal T-cell populations that produce eosinophil-promoting cytokines [17]. In contrast to CEL, the BM of patients with iHES appears normal with age-adjusted cellularity and regular eosinophils with bilobated nuclei [22, 59]. However, a subset of eosinophils may also display slight irregularities such as uneven cytoplasmic granulation and hypersegmentation [59]. As opposed to patients with MLN-TK, increased levels of serum tryptase are usually absent in iHES thus making it suitable as a rapid and cost-effective screening tool. The presence of genetic alterations should be ruled out by molecular studies (including PCR for the most frequent aberrations such as *FIP1L1:PDGFRA*, FISH analysis for other recurrent fusion genes and PCR for *KIT* D816V and *JAK2* V617F). If negative, NGS with the caveat of CHIP mutations (e.g., *DNMT3A*, *TET2* and *ASXL1*, usually present as single mutations with a low variant allele frequency) or RNA-sequencing might be applied in patients highly suspicious of clonal disease [2, 4, 59, 68].

**Table 5:** Diagnostic criteria for chronic eosinophilic leukemia (CEL) as per International Consensus Classification [4]

1. Peripheral blood hypereosinophilia (eosinophil count ≥ 1.5 × 10^9^/L and eosinophils ≥ 10% of white blood cells)
2. Blasts constitute < 20% cells in peripheral blood and bone marrow, not meeting other diagnostic criteria for AML^***a***^
3. No tyrosine kinase gene fusion including *BCR::ABL1*, other *ABL1, PDGFRA, PDGFRB, FGFR1, JAK2,* or *FLT3* fusions
4. Not meeting criteria for other well-defined MPN; chronic myelomonocytic leukemia, or SM ^***b***^
5. Bone marrow shows increased cellularity with dysplastic megakaryocytes with or without dysplastic features in other lineages and often significant fibrosis, associated with an eosinophilic infiltrate or increased blasts ≥ 5% in the bone marrow and/or ≥ 2% in the peripheral blood
6. Demonstration of a clonal cytogenetic abnormality and/or somatic mutation(s) ^***c***^

The diagnosis of CEL requires all 6 criteria

^***a***^ AML with recurrent genetic abnormalities with < 20% blasts is excluded

^***b***^ Eosinophila can be seen in association with SM. However, “true” CEL may occur as systemic mastocytosis associated with a myeloid neoplasms

^***c***^ In the absence of a clonal cytogenetic abnormality and/or somatic mutation(s) or increased blasts, bone marrow findings supportive of the diagnosis will suffice in the presence of persistent eosinophilia, provided other causes of eosinophilia having been excluded

The clinical presentation of iHES is heterogeneous and varies in the pattern and extent of single, multi-organ and potentially life-threatening organ damage. In cases with multi-organ involvement (e.g., lung, cardiac, gastrointestinal, cutaneous manifestations), the main differential diagnosis is EGPA. iHES or ANCA-negative EGPA can lead to cardiac involvement in up to 60% of affected patients, thus having a potential impact on morbidity and mortality [69–72]. Cardiac involvement often occurs early in the course of iHES or EGPA (predominantly in ANCA-negative EGPA patients) [9–13]. The onset of restrictive cardiomyopathy is associated with early death [73]. Clinical examination, electrocardiography, and cardiac biomarkers like N-terminal prohormone B-type natriuretic peptide (NT-proBNP) and troponin I provide important information. While conventional transthoracic echocardiography and endomyocardial biopsy have long been recognized as the standard diagnostic procedures, their sensitivity for early detection of myocardial infiltration/fibrosis is limited [71, 74–76]. Cardiac magnetic resonance imaging (MRI) has emerged as a valuable alternative imaging modality, providing non-invasive assessment of both structural and functional changes. In a study involving 62 patients with iHES, abnormal findings of cardiac MRI were correlated with elevated cardiac biomarkers and the pattern of organ involvement, indicating a risk of life-threatening cardiac events. Over a median follow-up of 108 months, 24% of patients experienced cardiac events, with the majority showing abnormal cardiac MRI and elevated biomarkers [77]. Accurate diagnosis of iHES, with confirmed exclusion of clonal eosinophilia, usually indicates a less aggressive disease course, with mortality rates ranging from 10–15% according to historical cohorts [59, 78–80].

Systemic corticosteroids are pivotal in treating patients with iHES [81]. Their usage is also highly informative for the distinction between reactive and clonal eosinophilia as there is usually no sustained effect of steroids on eosinophil counts in clonal disease. However, despite their effectiveness in responsive patients, prolonged use of high doses of systemic corticosteroids is often limited by severe adverse effects and long-term consequences. Therefore, the treatment goal is to gradually taper corticosteroids to a dose < 7 mg (prednisolone equivalent) per day. In cases of aggressive disease progression affecting multiple organs, a high "Five-factor-score" or resistance to systemic corticosteroids, early initiation of additional immunomodulatory agents like cyclophosphamide is highly recommended following re-evaluation for eosinophil clonality.

In a randomized, multicenter, double-blind, placebo-controlled, phase 3 trial, the treatment of iHES patients with mepolizumab (300 mg subcutaneously every month for a total of 32 weeks) significantly reduced the occurrence of flares (defined as worsening of HES-related symptoms necessitating therapy escalation or ≥ 2 courses of blinded rescue oral corticosteroids) and fatigue [82]. The open-label extension study (300 mg subcutaneously every month for a total of 20 weeks) demonstrated a continued control of disease flares (annualized flare rate 0.14 vs. 0.37 for placebo), a mean reduced corticosteroid dose ≥ 50% in 28% of patients and stabilized reduced blood eosinophil counts [83]. The most frequently reported flare symptoms were constitutional (94% of flares), dermatological (82% of flares) and respiratory (72% of flares) [84]. Symptom improvement was seen across all symptom groups except for skin and was highest for breathing symptoms [85]. A post hoc analysis revealed that treatment of mepolizumab was effective (flare outcome, symptom burden) independently of baseline eosinophil counts (minimal eosinophil count 1 × 10^9^/L) and IL-5 levels [86]. Furthermore, poorly controlled symptoms in patients with iHES could be alleviated irrespective of baseline therapy [87].

Benralizumab, a monoclonal antibody against the IL-5 receptor (IL5R) which is expressed by eosinophils, showed promising results in a randomized, double-blind, placebo-controlled, phase 2 trial when administered in a series of three-monthly subcutaneous injections at a dose of 30 mg. Benralizumab met the primary end point with at least 50% absolute eosinophil count reduction at week 12 in 90% of patients (vs. 30% for placebo). Subsequent open-label phase findings showed clinical and hematologic responses in 89% of patients. Notably, 64% of patients were able to reduce background therapies including systemic corticosteroids [88]. Benralizumab is currently undergoing a multicenter, randomized, double-blind, placebo-controlled, 24-week phase 3 study with an open-label extension (NCT04191304). Primary endpoint is the time to first HES worsening/flares. Benralizumab is approved as an add-on maintenance treatment in patients with severe eosinophilic asthma inadequately controlled despite high-dose inhaled corticosteroids plus long-acting β-agonists [89, 90].

Depemokimab is an anti-IL-5 monoclonal antibody that is currently studied in a multicenter, randomized, double-blind, placebo-controlled, 52-week phase 3 study (NCT05334368). It is administered subcutaneously every 26 weeks at a dose of 200 mg as a result from an encouraging phase 1 study in patients with asthma, where it showed an extended half-life, supporting less frequent dosing. [91]. As primary outcome, frequency of HES flares is measured. Consequently, the non-inferiority of switching participants who have benefitted from mepolizumab or benralizumab to depemokimab will explicitly be assessed in another ongoing clinical trial (NCT04718389).

Reslizumab, an anti-IL-5 monoclonal antibody, was investigated in four individuals with iHES [92, 93]. Patients were administered a single intravenous dose of 1 mg/kg. Following drug administration, two patients exhibited a favorable response with a decrease in PB eosinophils to normal levels within 48 h; eosinophil counts remained suppressed for up to 12 weeks post-treatment. The response was independent of IL-5 levels. In another study involving ten patients with EGPA, reslizumab (at a dose of 3 mg/kg) led to a significant reduction in daily oral corticosteroid usage [94]. On basis of two phase 3 trials, reslizumab was approved for add-on therapy in patients with severe eosinophilic asthma inadequately controlled despite high-dose inhaled corticosteroids plus another medicinal product for maintenance treatment (NCT01287039, NCT01285323) [95]. No clinical trial for evaluation in patients with iHES is currently listed for reslizumab. However, considering its intravenous administration based on body weight, as opposed to other anti-IL5(R) monoclonal antibodies, this drug may hold promise for patients with high BMI who have not responded to previous therapies.

Dexpramipexole, a synthetic aminobenzothiazole, was administered to 10 patients with iHES at a dose of 150 mg orally twice daily [96]. Results showed that 40% of patients experienced a reduction of ≥ 50% in their corticosteroid dose, while 30% exhibited a decline in PB eosinophils to < 0.01 × 10^9^/L with a depletion of eosinophils in the BM. Currently, there is no ongoing clinical trial enrolling patients with iHES.

Lirentelimab, a monoclonal antibody targeting sialic acid-binding immunoglobulin-like lectin (Siglec)-8, has been studied in various conditions including allergic conjunctivitis, chronic spontaneous/inducible urticaria, eosinophilic gastritis/duodenitis and indolent systemic mastocytosis [97–100]. These studies have demonstrated significant reductions in eosinophils in both PB and gastrointestinal tissue. Consistent with these findings, patients with ISM treated with lirentelimab showed rapid and specific decreases in eosinophil counts within one day of treatment, which were sustained throughout the 30-day treatment period. Currently, there is no ongoing clinical trial enrolling patients with iHES.

### Navigating future challenges and directions

Eosinophilic disorders encompass a spectrum of conditions ranging from reactive causes to clonal neoplasms. One pivotal diagnostic challenge lies in distinguishing between patients with iHES and ANCA-negative EGPA given the overlapping clinical, radiologic, and histologic features, and biomarker profile [101–104]. While the "5-Factor-Score" serves as a validated tool for predicting outcomes and guiding treatment approaches in EGPA, biomarkers predicting disease activity and prognosis remain largely elusive in iHES. The range (single vs. multiple organ involvement) and severity (constitutional versus cardiac symptoms) of possible organ manifestations complicates accurate assessment of overall disease activity as no specialized clinical, radiological and pathological assessment tools are currently defined. To date, primary endpoints in clinical settings frequently involve assessing the frequency of flares defined by worsening of HES-related symptoms necessitating therapy escalation, such as systemic corticosteroid administration. Evaluation based solely on blood eosinophil counts and tissue eosinophil infiltration is constrained, as symptoms and active disease complications may persist despite absence of eosinophilia, and validated thresholds for tissue eosinophilia remain lacking across most organ systems. Moreover, the absence of patient-reported outcome instruments validated for application in iHES limits the comprehensive assessment of health-related quality of life. With an expanding landscape of (approved) novel treatment options, further research regarding the optimal selection of initial therapy, potential factors contributing to treatment failure, and alternative therapeutic options for patients encountering incomplete response or disease progression is warranted.

With the advent and widespread application of NGS studies, individuals previously categorized as iHES may also undergo reclassification based on the mutational profile. However, expensive molecular analyses may be dispensable in clinically clear reactive cases (e.g., rapid remission on oral steroids) and a thorough interpretation of mutational alterations is warranted with the caveat of CHIP mutations as confounding factors [26]. Future priorities for patients with clonal eosinophilia will include the need for standard response criteria, the incorporation and harmonization of standard cytogenetic/FISH and molecular monitoring of fusion genes into clinical decision-making, refining dosing regimens, and exploring novel therapeutic modalities such as hypomethylating agents in CEL [21].

## Conclusions

In summary, our manuscript offers a thorough exploration of the evolving therapeutic options for eosinophilic disorders, covering established treatments as well as novel agents under investigation in clinical trials in reactive, clonal and idiopathic eosinophilic disorders. While patients with *PDGFRA* and *PDGFRB* fusion genes, who are receiving imatinib treatment, generally have a favorable prognosis, the advent of FGFR1 inhibitors offers hope for individuals with clonal eosinophilia and *FGFR1* fusion genes. New IL-5(R) antibodies mitigate morbidity and, consequently also mortality in cases of secondary and idiopathic eosinophilia. It is to emphasize that diagnosis and treatment of eosinophilic disorders rely on a multidisciplinary approach. Fostering collaborative research endeavors and interdisciplinary partnerships will help to gain new insights into the pathogenesis, diagnosis, and management of these complex disorders.

## Data Availability

No datasets were generated or analysed during the current study.
